# Relationship between social network, social support and health behaviour in people with type 1 and type 2 diabetes: cross-sectional studies

**DOI:** 10.1186/s12889-016-2819-1

**Published:** 2016-02-29

**Authors:** Nana F. Hempler, Lene E. Joensen, Ingrid Willaing

**Affiliations:** Steno Diabetes Center, Health Promotion Research, Niels Steensens Vej 8, 2820 Gentofte, Denmark

**Keywords:** Type 1 diabetes, Type 2 diabetes, Gender, Social network, Social support, Health behaviour

## Abstract

**Background:**

Psychosocial and behavioural aspects of diabetes may differ according to diabetes type. This study compared people with type 1 and type 2 diabetes with respect to social relations (cohabitation status, contact with the social network and social support) and health behaviours (diet and physical activity). Furthermore, we examined whether potential differences in health behaviour between people with type 1 and type 2 diabetes were influenced by education level and social relations.

**Methods:**

We conducted two cross-sectional surveys consisting of people with type 2 diabetes (*N* = 1081) and type 1 diabetes (*N* = 2419) from a specialist diabetes clinic. Gender-stratified stepwise multiple regression models assessed differences by diabetes type and other variables of interest.

**Results:**

Significant associations were found between diabetes type and social network, social support and health behaviour. No differences were observed regarding cohabitation status. People with type 2 diabetes were less physically active, less likely to follow recommended diet (men), had fewer contacts with family and friends and were less certain of counting on help in case of severe illness than people with type 1 diabetes. No impact of education level, social network and social support were observed concerning differences in health behaviours by diabetes type; however, in women, the association between physical activity and diabetes type was not significant after adjustment for social relations and education level.

**Conclusions:**

People with type 2 diabetes had less contact with the social network, less certainty about support in case of severe illness and fewer healthy behaviours than people with type 1 diabetes. It may be important to draw attention to differences in health behaviours and social relations between people with type 1 and type 2 diabetes in diabetes care, patient education and support initiatives.

## Background

Social networks and good social support are associated with better functioning, fewer psychosocial problems and improved diabetes self-management in people with diabetes [1–6]. Physical activity and healthy diet are recommended components of diabetes management in people with type 1 and type 2 diabetes [7–10]. Social support has been shown to be positively related to health behavioural change in chronic illness management, particularly in the field of diabetes [1]. While few studies shed light on differences between people with type 2 diabetes and the general population with regards to health behaviour and social relations (social support and social network contacts), little attention has been paid to possible differences between adults with type 1 and type 2 diabetes [11, 12].

Type 1 and type 2 diabetes differ substantially in relation to pathogenesis, symptoms, onset, cause of condition, treatment, complications, prognosis, etc. Diagnosis of type 1 and type 2 diabetes is expected to entail major changes in daily life and may affect social life in different ways. Type 2 diabetes often occurs later in adult life, so managing diabetes with respect to social life and changing health behaviour might be different for adults with type 2 diabetes compared with people with type 1 diabetes, where a strict care routine may have been introduced from a young age.

A more nuanced picture of people with type 1 and type 2 diabetes is required regarding possible differences in health behaviours and the impact of social relations on health behaviours. Moreover, the role of education level is an important component to consider when comparing social relations and health behaviours by diabetes type, as a poor social network and low social support are also more prevalent among people with low education compared to people with high education [13–15]. Studies have shown a similar pattern with regards to physical activity and dietary habits [16–18].

Many support initiatives and interventions focusing on behavioural and psychosocial aspects of diabetes do not differentiate by diabetes type [19, 20]. Insights into differences in social relations and health behaviour according to diabetes type are likely to be valuable for tailoring interventions and diabetes care. The purpose of this study is to: 1) compare social relations (cohabitation status, contacts with family, contacts with friends, perceived support from the social network) and health behaviours (diet and physical activity) of people with type 1 diabetes and type 2 diabetes; and 2) to explore to what extent associations between health behaviour and diabetes type are influenced by social relations and education level.

## Methods

We used the cross-sectional research design in two surveys, selecting 3500 people from Steno Diabetes Center, a specialist clinic in the Copenhagen area in Denmark (people with type 1 diabetes; ***N*** **=** 2419 and people with type 2 diabetes; ***N*** **=** 1081). The methods in the survey conform to the Declaration of Helsinki criteria and the studies were approved by the Danish Data Protection Agency (2011-41-6443 and 2010-41-4863).

### Steno Diabetes Center: population with type 1 diabetes

In 2011, 3626 people with type 1 diabetes from the center received a letter with a questionnaire concerning self-management behaviours, social networks, health, and psychosocial aspects such as diabetes distress and social support [21]. The letter also included a prepaid envelope. Two weeks after the questionnaire was sent out, the first reminder was sent to non-respondents, followed by a second reminder at four weeks. Both reminders included a new questionnaire and a prepaid envelope. An email and telephone service operated by a diabetes nurse was available for patients during the data collection. Data collection ended after 6 weeks.

### Steno Diabetes Center: population with type 2 diabetes

In 2010, 2045 people with type 2 diabetes received a letter with a questionnaire focusing on preferences for patient education, social networks and self-management behaviours [22]. The letter contained information about the survey and a web address for the online questionnaire. Two weeks after the first mailing a reminder was sent. Two weeks after the reminder was mailed, a third letter was mailed to those who had not completed the questionnaire, which included a paper version of the questionnaire and a prepaid return envelope. Data collection ended after 6 weeks.

### Measurements

We included validated questions about health behaviour, education level, social support and social network in both questionnaires. We used two items from a validated revised version of the Summary of Diabetes Self-care Activities measure to assess health behaviour related to physical activity and diet [23]. The items assessed general diet and general exercise: ‘On how many of the last seven days did you participate in at least 30 min of physical activity?’ and ‘How many of the last seven days have you followed a healthful eating plan’? Information concerning education level was measured in a single item: 1) primary and lower secondary education, 2) upper secondary and vocational school, 3) medium higher education (3–4 years), 4) long higher education (≥5 years). Data on sociodemographic data (sex and age) and diabetes type were obtained from the electronic patient record at Steno Diabetes Center.

We distinguished social relations by structure (social network) and function (social support), as described by Due et al. [24]. To measure social relations, we used validated questions from the Danish Health and Morbidity Survey [25]. Questions about social network included: 1) cohabitation status (living without a partner or spouse, yes/no); 2) frequency of contacts with friends (almost every day/once or twice a week/once or twice a month vs. less than once a month/never); 3) frequency of contacts with family (almost every day/once or twice a week/once or twice a month vs. less than once a month/never). Perceived social support was measured by a validated item from the Danish Health and Morbidity Survey, assessing to what extent participants were certain of counting on help in case of severe illness (definitely/maybe vs. no) [25].

### Statistical analysis

Using logistic regression models, we compared social network and social support by diabetes type. Initially, we explored if education level and gender might modify the effect of the association between diabetes type and social relations by calculating the relative excess risk due to interaction (RERI), using a method outlined by Anderson et al. [26]. We applied this method because interpretation of interaction effects in logistic regression models differs from linear regression models [27]. In accordance with the method, education level was dichotomized into primary/lower secondary education and upper secondary/medium/higher education. In case of no positive or negative RERI scores with associated confidence intervals above or below 0, we included education level and gender as a possible confounder in the models. All analyses were adjusted for age and diabetes duration. Additional covariates were added in the models based on assumptions of causal relations. For example, cohabitation status was assumed to influence contacts with the social network and therefore we adjusted for cohabitation status when we explored the association between diabetes type and social network. Furthermore, perceived social support was expected to be influenced by contact with social network and analyses exploring the association between diabetes type and social support were adjusted for social network variables.

Generalised linear regression models with 95 % confidence intervals (CI) were applied to analyse the association between diabetes type and diet/physical activity. Initially, we explored interaction terms between education level and diabetes type and between gender and diabetes type with respect to health behaviour. If interaction terms were significant (*P* < .05), analyses were stratified according to the specific variable. If interaction terms were non-significant, we included education level and gender as covariates in the models. Social network variables and social support variables were included stepwise in the models based on assumptions of causal relations. If education level and social network variables were significantly associated with the variable of interest, they were included in subsequent steps.

All analyses were adjusted for age and diabetes duration. Data were analysed using SAS statistical version 9.2 (SAS Institute Inc., Cary, NC, USA).

## Results

### Participants and response rates

In the population with type 1 diabetes, 21 people were excluded because they were unable to be reached and 14 people had died or reported that they did not have type 1 diabetes. Of the remaining 3591 people, 2419 completed the survey, corresponding to a response rate of 67 %. Non-respondents were younger, consisted of fewer women, had higher HbA1c and longer duration of diabetes, compared with respondents. In the population with type 2 diabetes, 59 people were excluded because they had died, had dementia or reported that they did not have diabetes. One thousand eighty-four answered the questionnaire, corresponding to a response rate of 54.0 %. Non-respondents were older, consisted of more women and had higher HbA1c whereas diabetes duration did not differ significantly, compared with respondents.

### Socio-demographic characteristics (gender, age, education level and diabetes duration)

Men were overrepresented among people with type 2 diabetes, compared to people with type 1 diabetes (Table 1). People with type 1 diabetes were generally younger, had longer diabetes duration. People with type 2 diabetes had a lower education level than people with type 1 diabetes (Table 1).

**Table 1:** Characteristics by diabetes type

	Type 1 diabetes (*N* = 2419)	Type 2 diabetes (*N* = 1081)	*P*-value
Gender (%)			
Men	52.0	65.5	<0.0001
Age (%)			
16–24 years	5.4	0.1	<0.0001
25–34 years	9.6	0.7	
35–44 years	17.1	4.6	
45–54 years	23.4	13.0	
55–64 years	22.7	30.1	
65–74 years	15.7	33.7	
>75	6.2	17.9	
Missing	0.1	0	
Mean diabetes duration (years)	26.9	14.8	<0.0001
Missing	2.9	2.6	
Education level (%)			
Primary and lower secondary education	15.5	21.8	<0.0001
Upper secondary or vocational education	35.4	42.7	
Medium education	22.6	14.7	
Long higher education	18.4	14.7	
Missing	8.2	6.0	
Physical activity at least 30 min. (mean days in a week)	4.74	4.11	<0.0001
Missing	3.9	11.4	
Eating health foods (mean days in a week)	5.92	5.12	0.0001
Missing	6.8	11.1	
Cohabitation status (%)			
Living without a partner	29.8	29.4	0.9313
Missing	2.4	4.4	
Meet with family less than once a month (%)	6.2	12.0	<0.0001
Missing	2.1	4.3	
Meet with Friends less than once a month (%)	5.4	10.6	<0.0001
Missing	2.2	3.9	
Not certain of counting on help in case of severe illness	17.6	25.6	
Missing	1.8	3.8	<0.0001

*Chi-square tests, *t* tests or Wilcoxon tests as appropriate

### Initial analyses of education level and gender as possible effect modifiers in the models

Initially, we tested education level and gender as possible effect modifiers in relation to all outcomes. When testing outcomes of social relations we used the method by Anderson et al. We found no strong indication for education level as an effect modifier of the association between diabetes type and social relations (RERI scores between −0.60 and 0.21 and all confidence intervals included 0), and, as a result, education level was included as a covariate in subsequent logistic regression analyses. Regarding gender, analyses suggested that gender was an effect modifier in the association between diabetes type and social relations, and the logistic regression analyses were stratified according to gender (RERI scores between −1.48 and 1.64 where most confidence intervals were above or below 0).

In generalised linear regression models, no significant interaction terms were found between diabetes type and education level with regard to diet (*p* = 0.7339) or physical activity (*p* = 0.2109), and we consequently included education level as a covariate in the analyses. The p values for the interaction term for gender and diabetes type in relation to diet and physical activity were respectively 0.0050 and 0.0017, and analyses were stratified according to gender.

### Social relations (social network and social support)

For both genders, we found no differences in regard to cohabitation status by diabetes type, also after adjustment for education level (Table 2). With respect to contacts to the network, the overall pattern was that men and women with type 2 diabetes had less contact with family and friends, compared with men and women with type 1 diabetes. Differences between people with type 1 and type 2 diabetes were more pronounced among women than men. Furthermore, people with type 2 diabetes were less certain they could count on help in case of severe illness, compared to people with type 1 diabetes. For most outcomes, estimates attenuated slightly when education level and remaining social network covariates were included in the models (Table 2, Model 2–3).

**Table 2:** Odds ratio estimates for social network and social support by diabetes type, stratified by gender

	Type 2 diabetes	
	Men (OR)	Women (OR)
Living without a partner		
Step 1	0.90 (0.71–1.14)	1.26 (0.88–1.79)
Step 2	0.89 (0.69–1.13)	1.16 (0.80–1.68)
Meet with family less than once a month		
Step 1	1.76 (1.24–2.51)	3.57 (1.85–6.90)
Step 2	1.67 (1.17–2.39)	3.25 (1.60–6.59)
Step 3	1.57 (1.07–2.29)	2.59 (1.22–5.50)
Meet with friends less than once a month		
Step 1	2.70 (1.81–4.04)	4.99 (2.88–8.67)
Step 2	2.49 (1.65–3.74)	4.46 (2.51–7.95)
Step 3	2.35 (1.53–3.62)	3.95 (2.16–7.19)
Not sure they can count on getting help		
Step 1	1.40 (1.09–1.80)	2.82 (1.91–4.18)
Step 2	1.41 (1.09–1.83)	2.85 (1.89–4.29)
Step 3	1.31 (1.00–1.72)	2.31 (1.47–3.62)

Reference group: men or women with type 1 diabetes

Note: Logistic regression analyses including age and diabetes duration in all steps. Education level was included in step 2 and 3. Social network variables (living without a partner, contact with friends/family) were included in step 3

### Health behaviour (diet and physical activity)

Men with type 1 diabetes were more likely to eat a healthy diet compared with men with type 2 diabetes after adjustment for age and diabetes duration, whereas for women we found no significant differences (Table 3, Step 1). In men, the association between diabetes type and diet remained significant after adjustment for education level, social network variables and social support. In women, the association remained non-significant after adjustment for covariates. Only in men, we found a significant association between education level and dietary habits and between social support and dietary habits. In women, there was an association between dietary habits and cohabitation status as well as having contact with friends.

**Table 3:** Regression coefficients for health behaviour by diabetes type, stratified by gender

		Eating healthy foods (mean days in a week)				Physical activity at least 30 min. (mean days in a week)			
		Men		Women		Men		Women	
		β	p	β	p	β	p	β	p
Step 1	Type 2 diabetes	−0.46	<0.0001	−0.11	0.4145	−0.34	0.0079	−0.30	<0.0001
Step 2	Type 2 diabetes	−0.41	<0.0001	−0.03	0.8448	−0.34	0.0100	−0.49	0.0204
	Primary/lower secondary education	−0.68	<0.0001	−0.16	0.2934	0.07	0.7071	−0.43	0.0624
	Upper secondary education	−0.43	<0.0001	−0.12	0.3672	0.04	0.7608	−0.19	0.3326
	Medium education	−0.30	0.0211	−0.02	0.9011	0.11	0.5442	0.26	0.2235
Step 3	Type 2 diabetes	−0.43	<0.0001	−0.07	0.6221	−0.30	0.0225	−0.23	0.2817
	Living without a partner	0.04	0.6702	−0.34	0.0002	0.01	0.9391	−0.38	0.0054
	Limited contact with family	−0.04	0.7664	−0.18	0.3297	0.24	0.2313	−0.63	0.0440
	Limited contact with friends	−0.37	0.0214	−0.47	0.0091	−0.96	<0.0001	−1.48	<0.0001
Step 4	Type 2 diabetes	−0.43	<0.0001	0.08	0.5769	−0.33	0.0109	−0.22	0.3000
	Low social support	−0.14	0.0120	−0.14	0.2424	−0.21	0.2164	−0.03	0.8685

Reference group in all analyses: men or women with type 1 diabetes

Note: Generalised linear regression models included age and diabetes duration in all steps. Education level was included in step 2 and in 3 and 4 if significant. Social network variables were included in step 4 if significant. Beta parameters not shown

Regarding physical activity, people with type 2 diabetes were less physically active than people with type 1 diabetes (Table 3, Step 1). Associations remained significant after adjustment for education level. Differences by diabetes type were less pronounced in men after adjustment for social network covariates but remained significant (step 3). In women, the association between diabetes type and physical activity was no longer significant after adjustment for social network variables. Social support and education level were not associated with physical activity for both men and women.

## Discussion

Our findings suggest that, compared to men and women with type 1 diabetes, men and women with type 2 have less contact with family and friends and are less certain about support in case of severe illness. Differences were more pronounced among women than in men. Men with type 2 diabetes had worse dietary habits than men with type 1 diabetes, and associations were not influenced by education level, social network contacts or social support. In women, we found no differences in dietary habits according to diabetes type. Furthermore, men and women with type 2 diabetes reported lower physical activity levels than men and women with type 1 diabetes. In women, social network contacts seemed to explain differences in physical activity according to diabetes type.

To be best of our knowledge, only one study has compared social relations and health behaviour with respect to diabetes type. The study by Aalto et al. showed that men with type 2 diabetes were more likely to be single/unmarried and to report less contact with friends than men with type 1 diabetes, whereas no differences were observed for women [11]. However, in our study we found similar patterns for men and women regarding cohabitation status and contact with friends. Variations in findings across studies may be a result of differences in how social variables were measured and how analyses were performed.

Alto et al. found better dietary habits (regular meals) among people with type 1 diabetes, compared with those with type 2, particularly among men, as we did in our study. Furthermore, Aalto et al. also found that men with type 1 diabetes reported more exercise than did men with type 2 diabetes, whereas differences were less pronounced in women [11].

The fact that people with type 2 diabetes reported fewer social relations than people with type 1 diabetes may be explained by several factors. Older age and possibly poorer lifestyle among people with type 2 diabetes may be associated with comorbidities such as cardiovascular diseases, chronic obstructive pulmonary disease and arthritis, all factors likely to increase the need for social support and making it difficult to establish, maintain and strengthen contacts with the social network. Another possible explanation for fewer social relations and poorer health behaviours among people with type 2 diabetes could suggest a reverse causation, as lack of social support and social network may promote stress and poorer health behaviour and thereby increase the likelihood of type 2 diabetes.

People with type 2 diabetes reported poorer health behaviours in terms of dietary habits and physical activity than did those with type 1 diabetes, with the exception of dietary habits in women. This was expected due to the relationship between type 2 diabetes and health behaviours. Notably, social relations only diminished the association between diabetes type and of physical activity in women slightly, underscoring the importance of addressing gender differences. In addition, it is also known from the literature that women generally have more and closer contact with a social network than do men [24, 28].

Regarding the role of education, our findings did not suggest education level as an effect modifier of the observed associations. Education level as a covariate had no strong effect with respect to social relations. However, education level seemed to play a role regarding diabetes type differences in physical activity. Regarding diet, we found that education level was highly associated with diet but did not influence the association between diabetes type and diet. This might be explained by the fact that differences in education level in our data were less pronounced in men compared with women.

Strengths of our study include that we surveyed two large populations from the same area. Survey data were merged with data from an electronic patient record which allowed comparison of respondents and non-respondents on selected variables. Limitations include the cross-sectional design which prevented us from determining causal relationships between diabetes type and the outcome variables and the influence of education level. Differences in the participation rate and characteristics of respondents and non-respondents differed by diabetes type and may cause some bias. Non-respondents in the type 1 population were younger, consisted of fewer women, and had shorter diabetes duration whereas this was reversed in the type 2 population. However, in both populations, non-respondents had higher HbA1c levels than respondents. In addition, the higher participation rate in the type 1 population might be influenced by differences in education and literacy, which are expected to be higher in the type 1 population compared to the type 2 population. Furthermore, perceived social support was measured by a single variable; according to Due et al., it is multidimensional, including social support, relational strain and social anchorage [24]. In addition, social support can also be investigated in terms of positive vs. negative, perceived available vs. perceived received and directive vs. non-directive [29].

## Conclusions

Effective development of interventions incorporating behavioural and social aspects of living with diabetes requires a broad perspective, including an understanding of associations between social relations and diabetes type and on the influence of social relations on health behaviour for people with type 1 and type 2 diabetes. Our findings suggest that people with type 2 diabetes have less contact with the social network, less certainty about support in case of severe illness and fewer healthy behaviours than people with type 1 diabetes. Furthermore, for women, having a good social network and receiving social support appear to be associated with being more physical active. In clinical practice, paying attention to differences between people with type 1 and type 2 diabetes with regard to social relations and health behaviours may be important when planning patient care and support. More research is needed, particularly on how to support people with type 1 and type 2 diabetes, few social network resources and low education level in relation to disease management.
